# Highly efficient photoelectric effect in halide perovskites for regenerative electron sources

**DOI:** 10.1038/s41467-021-20954-6

**Published:** 2021-01-29

**Authors:** Fangze Liu, Siraj Sidhik, Mark A. Hoffbauer, Sina Lewis, Amanda J. Neukirch, Vitaly Pavlenko, Hsinhan Tsai, Wanyi Nie, Jacky Even, Sergei Tretiak, Pulickel M. Ajayan, Mercouri G. Kanatzidis, Jared J. Crochet, Nathan A. Moody, Jean-Christophe Blancon, Aditya D. Mohite

**Affiliations:** 1 Los Alamos National Laboratory, Los Alamos, NM USA; 2 Department of Chemical and Biomolecular Engineering Rice University, Houston, TX USA; 3 Department of Material Science and Nanoengineering Rice University, Houston, TX USA; 4 Univ Rennes, INSA Rennes, CNRS, Institut FOTON - UMR 6082, 20, Avenue des buttes de Coesmes, Rennes, France; 5 Department of Chemistry, Northwestern University, Evanston, IL USA; 6 Department of Materials Science and Engineering, Northwestern University, Evanston, IL USA; 7 Argonne-Northwestern Solar Energy Research (ANSER) Center, Northwestern University, Evanston, IL USA

**Keywords:** Semiconductors, Materials for devices, Electronics, photonics and device physics

## Abstract

Electron sources are a critical component in a wide range of applications such as electron-beam accelerator facilities, photomultipliers, and image intensifiers for night vision. We report efficient, regenerative and low-cost electron sources based on solution-processed halide perovskites thin films when they are excited with light with energy equal to or above their bandgap. We measure a quantum efficiency up to 2.2% and a lifetime of more than 25 h. Importantly, even after degradation, the electron emission can be completely regenerated to its maximum efficiency by deposition of a monolayer of Cs. The electron emission from halide perovskites can be tuned over the visible and ultraviolet spectrum, and operates at vacuum levels with pressures at least two-orders higher than in state-of-the-art semiconductor electron sources.

## Abstract (web summary)

Electron sources play as important component in a wide range of applications. Here, the authors demonstrate efficient, regenerative, and low-cost electron sources based on solution-processed halide perovskite thin films with quantum efficiency up to 2.2% and a lifetime of more than 25 h.

## Introduction

Halide perovskites are now recognized as a new class of semiconductors with attractive properties for applications in optoelectronics, energy technologies, and photonics^1,2^. The materials abundance and low cost, ease of fabrication of single crystals or thin films using low temperature (100 °C) solution processing, tunability of their optical and electronic properties, and ability to carry relatively large charge current without achieving the level of crystal quality required in classic inorganic semiconductors (e.g. III-V, CdTe, Cu(In,Ga)Se_2_) are the main reasons halide perovskites have drawn so much attention in the past few years. Although the main efforts in halide perovskite have been driven by record efficiencies in photovoltaics (>25%), this class of semiconductors has recently seen a surge in the development of efficient proof-of-concept devices for diverse applications including light-emitting diodes, scintillators, X-ray, and gamma radiation detectors^3–9^. Surprisingly, the figures of merit of halide perovskite-based devices have been on par with state-of-the-art inorganic semiconductor devices in several of these applications, which are consequences of the excellent intrinsic physical properties of halide perovskites as elucidated by several papers^10–13^.

One of the most fundamental and technologically relevant phenomena in light-matter interaction is the demonstration of the photoelectric effect, where photons have been used to emit electrons in free space. Efficient electron emission from various types of materials has enabled technologies for many cutting-edge applications such as free-electron lasers, image intensifiers for night imaging, sources for next-generation electron-beam lithography, electron microscopy, and photomultipliers; it has also led to the elucidation of novel materials physics probed through the investigation of the spin, momentum, and energy of the emitted electron^14–17^. State-of-the-art electron sources (or photocathodes) are classified into three families depending on the active material type: metals (Cu, Mg, Pb), alkali antimonide and tellurides (Cs_2_Te, K_2_CsSb, Cs_3_Sb), and III-V semiconductors (GaAs, GaN, tertiary alloys of III-V materials)^18^. The differences between these photocathodes pertain to their figures of merit including their quantum efficiency (QE, defined as the number of free electrons emitted per excitation photon) and lifetime (usually defined as the time for the QE to degrade to 1/e of its initial value). For example, while metal-based electron sources exhibit excellent lifetime in operation (several months), their QE is typically of the order of 0.1% or less, which is more than two orders of magnitude lower than state-of-the-art semiconductor-based electron sources. On the other hand, the latter type of electron sources is more sensitive to degradation, and requires intensive material and device preparation^18–21^. The main advantage of non-metallic photocathodes is that they generate high free-electron currents (several milliamperes) due to their high QE spanning the ultraviolet and visible light spectrum range. Typically, QE larger than 10% can be achieved in both alkali- and III-V electron sources of high quality with crystalline perfect surfaces and usually after surface treatment that allows tunneling of electrons through the surface barriers and into vacuum^18,21,22^. For example, GaAs photocathodes achieve high QE by means of negative electron affinity (NEA) at their surface equivalent to surface dipoles pointing to the material bulk, which requires the deposition of an atomically thin layer of strongly electropositive alkali metal (usually Cs) on their surface and subsequent exposition to an oxidizing agent^23,24^.

Specifically, the semiconductor-based electron source technologies share stringent requirements in terms of material quality and require operation in ultra-high vacuum (typically 10^11^–10^−12^ torr) with only traces of contamination elements such as H_2_O, O_2_, CO_2_^18,25–27^. The latter impacts mainly the lifetime of the devices while the former determines the figures of merit of the electron sources including their QE. Thus, acting on one or both these constraints by reducing the complexity of the materials preparation, integration, and surface cleaning or by being able to operate the electron source at a higher pressure of vacuum will accelerate the development of such technologies through reducing the cost of fabrication and operational facilities, as well as by enabling electron sources with new technical and operational specifications. All of these have motivated the investigation of new classes of semiconductors for their potential use as easily replaceable, efficient, durable, low-cost, scalable, and adaptable (size, shape, and substrate) electron sources.

Here, we invoke Einstein’s photoelectric effect, to report for the first time, efficient free-electron emission halide perovskite thin films operating in the visible to the ultraviolet spectral range. We measure a peak QE of 2.2% for the photoelectric effect and a lifetime exceeding 25 h under continuous operation and a vacuum pressure of 10^−9^ torr, which is more than two-orders higher than pressures typically used in state-of-the-art electron sources. We also show that electron emission can be completely regenerated to its maximum QE even after the perovskite electron source has almost completely degraded using an in-situ Cs deposition process. Finally, we show that by tuning the elemental composition of the halide perovskite films we can obtain electron emission across the entire visible spectrum. We anticipate that the observation of efficient electron generation using the photoelectric effect in halide perovskite thin films could pave the path for the development of a disruptive technology for realizing low-cost, high efficiency, and spectrally tunable electron sources based on halide perovskite semiconductors, which can be easily integrated and operated at much higher pressures of vacuum than their classic semiconductor-based counterparts without appreciable surface pre-treatments.

## Results

### Characterization of the photoelectric effect in halide perovskites

**Fig. 1:**
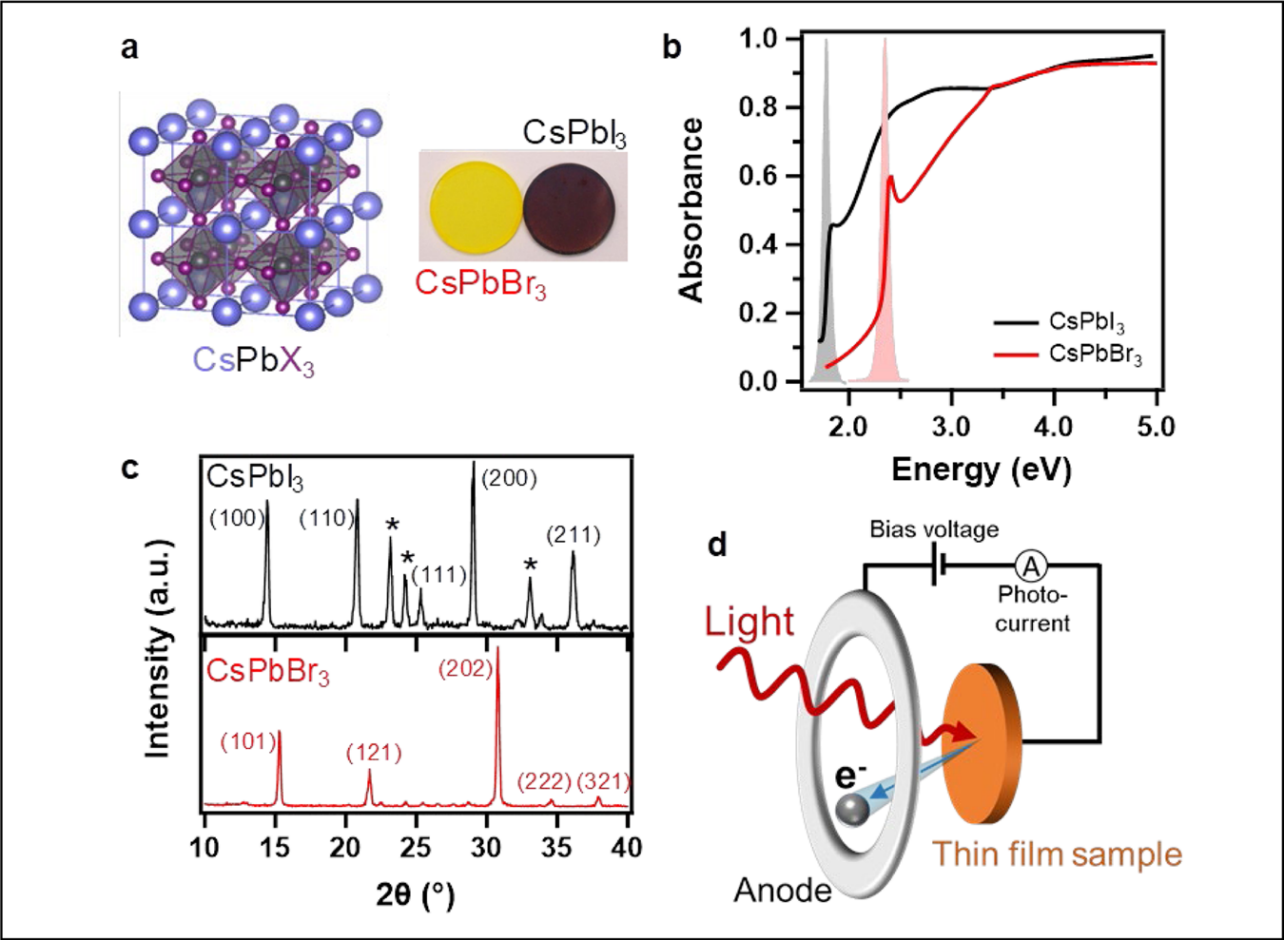
Characterization of the halide perovskite thin films. **a** Cubic structure of the halide perovskites and picture of thin films on glass substrates. **b** Absorbance and photoluminescence (gray and red) spectra of the CsPbI3 and CsPbBr3 thin films. **c** Corresponding X-ray diffraction spectra. The stars indicate the presence of a residual non-perovskite yellow phase in CsPbI3. a.u. denotes arbitrary units. **d** Schematic of the photoelectric effect measurement setup. The films were prepared on 1 inch^2^ silicon substrates for this measurement.

Halide perovskite thin films (CsPbX_3_, *X* = Br or I) were made by mixing precursor powders (PbX_2_ and CsX) with the desired molar ratio in dimethyl sulfoxide or N, N-dimethylformamide and spin-coating on doped Si wafers. The sample preparation was made in a standard glovebox under Ar atmosphere to limit air contamination of the film’s surface. The crystal structure of CsPbX_3_ along with the optical image of the films are presented in Fig. 1a. We characterized the absorbance, photoluminescence, and structure of both perovskite films in Fig. 1b, c (notice that CsPbI_3_ and CsPbBr_3_ respectively adopt the cubic and orthorhombic phases at room temperature) before loading them into an ultra-high vacuum chamber for photoelectric effect characterizations. The base pressure of the chamber was about 1 × 10^−9^ torr with contaminant gas partial pressure below 10^−11^ torr, and the films were annealed at 350 °C in the chamber to clean their surface from any organic or gas contaminant. We performed Auger electron spectroscopy (AES) on CsPbBr_3_ before and after annealing, as shown in Supplementary Fig. 7. The magnitudes of characteristic Auger peaks from Cs (49 eV), Br (55 eV), and Pb (97 eV) remained unchanged after UHV annealing. Moreover, we observe significant attenuation of the Auger signals associated with carbon (275 eV) and oxygen (510 eV). These results suggest that the chemistry of the perovskite thin films is not affected by UHV annealing while the latter process effectively removes oxygen adsorbed onto thin film surfaces, as well as amorphous carbon and solvent residue. The photoelectric effect was observed and studied by illuminating the sample with a monochromatic light beam and measuring the resulting photocurrent between the thin film sample and a counter anode while applying a potential difference between the two (Fig. 1d). The QE energy spectrum of the photoelectric effect was derived from the measurement of the photocurrent response as a function of the excitation light energy over the ultraviolet and visible range. The measurements were performed with and without the deposition in-situ of an ultra-thin layer (ideally one to a few monolayers) of Cs on the surface of the perovskite films.

**Fig. 2:**
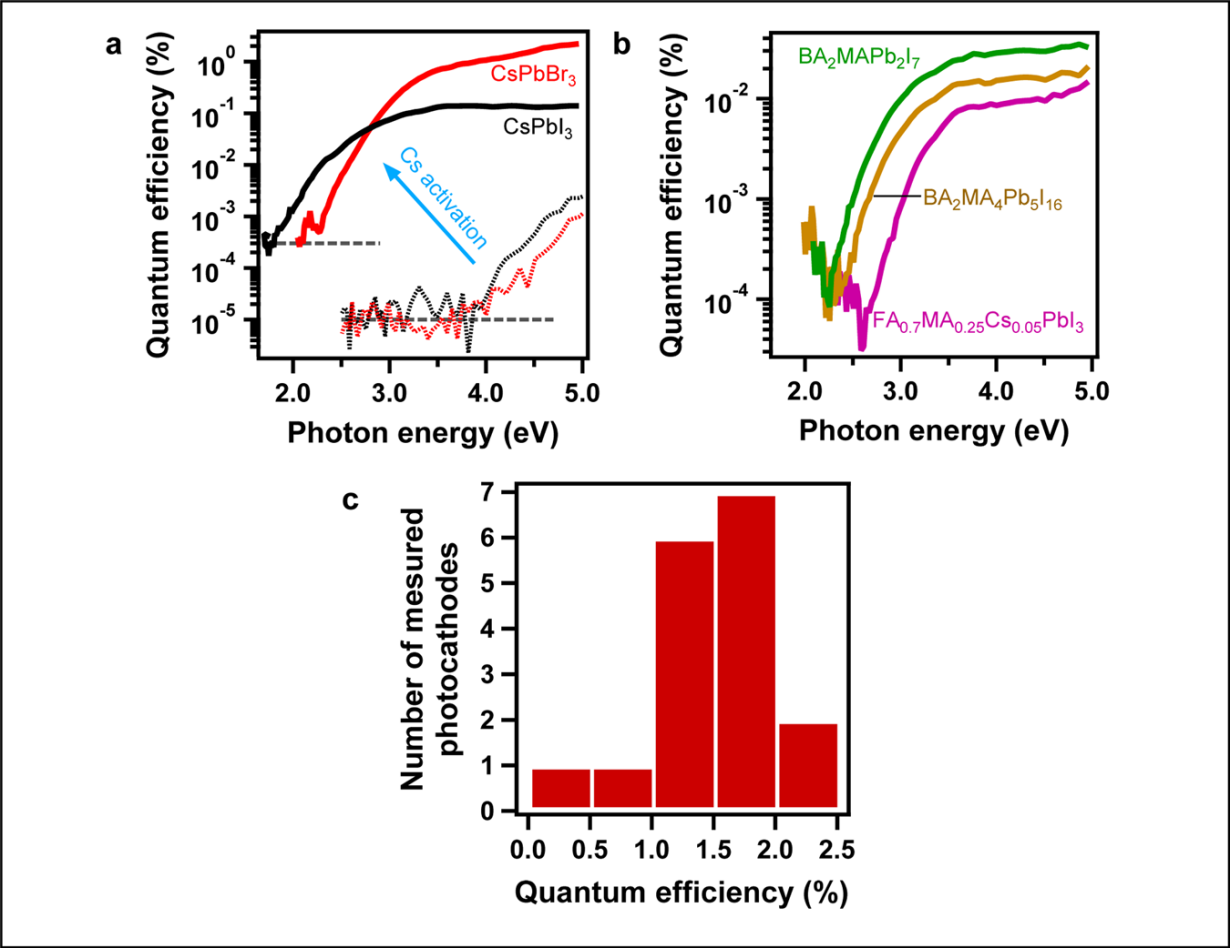
Efficiency of the photoelectric effect in halide perovskites. **a** Quantum efficiency spectra of the photoelectric effect in the CsPbI3 and CsPbBr3 thin films before (dashed lines) and after (solid lines) Cs activation. Dashed gray horizontal lines indicate the emission threshold. **b** Same for the hybrid perovskite films: FA0.7MA0.25Cs0.05PbI3, BA2MA1Pb2I7, and BA2MA4Pb5I16. **c** Statistics of the maximum quantum efficiency performance measured for seventeen CsPbBr3 thin films.

Figure 2a illustrates the QE spectrum of the photoelectric effect for the CsPbI_3_ and CsPbBr_3_ thin films measured before (dashed curves) and after Cs deposition (solid curves). We observe a dramatic three to four orders of magnitude increase in the QE for all photon energies after the Cs deposition. Concomitant with the increase in the QE, we observe a lowering of the onset energy of the electron emission (i.e. the minimum photon energy necessary to observe the photoelectric effect) from 4.0 eV to about the bandgap energy of CsPbBr_3_ (2.1 eV) and CsPbI_3_ (1.8 eV). The maximum QE values that were measured in the CsPbBr_3_ and CsPbI_3_ thin films are 2.2% at 5 eV and 0.14% at 3.5 eV, respectively. In the CsPbBr_3_ films, the QE value remains greater than 1% for UV light with energy above 3.9 eV, and was measured to be larger than 0.25% at 3.1 eV (400 nm). These values are one order of magnitude smaller than the QE reported in the state-of-the-art III-V semiconductor electron sources (e.g. GaN after Cs activation yields QE larger than 10% at 4.77 eV)^18,21,22^, which is rather impressive given the fact that III-V semiconductor electron sources involve complex and costly fabrication processes and surface pre-treatments in order to create high-quality materials with atomically perfect surfaces. In the case of halide perovskite thin films, the fabrication is simple and low cost, with the only demanding step being the Cs deposition. We also note that the QE performances of the halide perovskite films are more than two orders of magnitude better than metallic electron sources in the UV range and at a comparable vacuum level^19^. The reproducibility of the high QE observed in CsPbBr_3_ thin films was verified by testing 17 films yielding an average QE larger than 1.5% with two champion films exhibiting QE larger than 2% at 5 eV as illustrated in Fig. 2c.

Next, we tested whether the Cs surface activation process was applicable to the broad family of organic–inorganic (hybrid) halide perovskites. The latter materials contain extremely reactive organic salts such as methylammonium (MA), butylammonium (BA), or Formadinium (FA), which replace the Cs atoms in the perovskite lattice (Fig. 1a). Three distinct hybrid perovskites that are used for fabricating state-of-the-art perovskite-based photovoltaic cells were tested: the 3D perovskite FA_0.7_MA_0.25_Cs_0.05_PbI_3_^28^, and the Ruddlesden-Popper layered hybrid perovskites BA_2_MA_n-1_Pb_n_I_3n+1_ with *n* = 2 or 5^29^. Here, we note that the hybrid perovskites were not annealed in an ultra-high vacuum because annealing these materials above 100 °C is known to sublimate the organic cation and degrade the perovskites^30^. Despite these constraints, all the hybrid perovskite thin films demonstrated relatively efficient photoelectric effect with a low energy onset after Cs activation (Fig. 2b and Supplementary Fig. 1). The emission onset for BA_2_MA_1_Pb_2_I_7_ is close to its intrinsic bandgap, i.e. 2.35 eV (photoelectric onset) versus 2.18 eV (optical bandgap), where the difference between these two values could be accounted for by the binding energy of electron-hole pairs (excitons)^31^. However, the onset of electron emission was blue shifted in the other two hybrid perovskite thin films as compared to their bandgap energy. We speculate that this discrepancy is partly explained by our lack of control of the Cs activation process and the presence of absorbed gases on the film’s surface (no annealing was done for hybrid perovskites), both of which can significantly affect the electronic structure on the surface of the perovskite films. The QE of the hybrid perovskites is of the order of 0.01% for photon energy between 3 and 3.5 eV, which is two-orders of magnitude higher than metallic electron sources in this energy range^18^. These results attest to the robustness and potential of hybrid halide perovskites as electron sources despite the presence of reactive organic cations.

### **U**nderstanding the physical origin of the photoelectric effect after Cesium activation

**Fig. 3:**
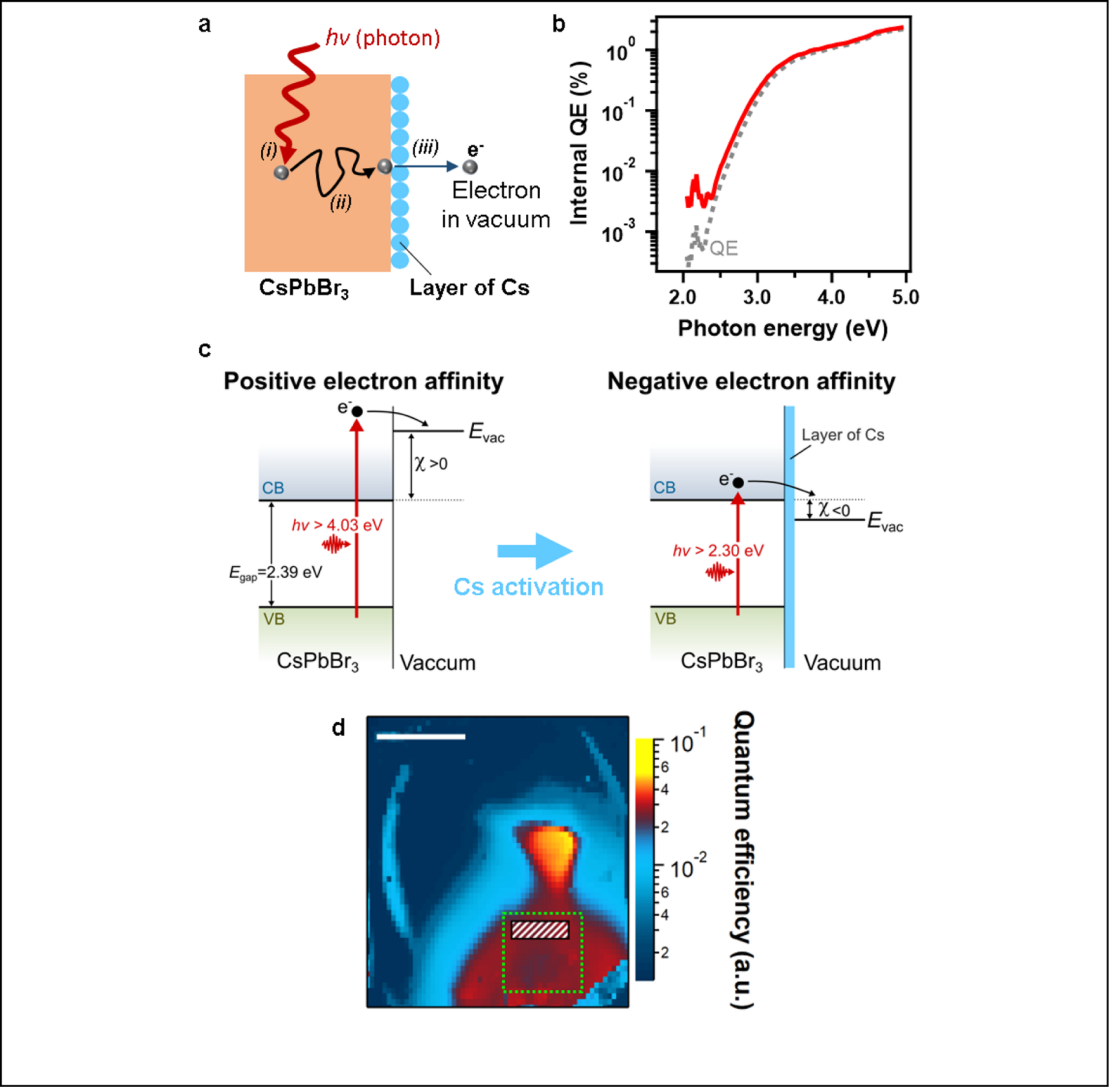
Physical origin of the efficiency of the photoelectric effect. **a** Mechanism of the photoelectric effect showing the three main steps: (i) electron photogeneration, (ii) electron transport, and (iii) electron emission. **b** Internal quantum efficiency of the photoelectric effect in the CsPbBr3 thin film after Cs activation. The gray dashed curve shows the corresponding (external) quantum efficiency, copied from Fig. 2a. **c** Schematics of the band diagram at the CsPbBr3 thin film surface before and after Cs activation. Egap, VB, CB, χ stand for the bandgap, the valence band, the conduction band, and electron affinity. Evac indicates the vacuum level where the electron can be emitted from the surface after photoexcitation (hν). **d** Spatial map of the quantum efficiency relative to its maximum value for the CsPbBr3 thin film after Cs activation. Scale bar is 10 mm. Dashed area mark the position of the in-situ Cs source. The quantum efficiency exhibits peak values near the center of the sample in a 10 mm^2^ area, while other regions of the samples yield slightly smaller but uniform efficiency within 10% margin variations over a 1 cm^2^ (indicated by dashed-green-square region).

To further understand what limits the QE and what defines the onset photon energy of the electron emission in the halide perovskite thin films, we investigated the mechanism of the photoelectric effect, which consists of three basic steps (Fig. 3a): (i) absorption of photons in the bulk of the halide perovskite films and generation of photo-excited electrons in the conduction band, (ii) transport of the photo-excited electrons to the films surface, and (iii) escape of these electrons to the vacuum by surface tunneling. We tested if the step (i) was limiting the QE of the perovskite thin films by calculating the internal quantum efficiency defined as the QE divided by the absorbance (Fig. 1c and Fig. 2a). It yielded nearly an identical response as the QE in all types of perovskites (Fig. 3b and Supplementary Fig. 2), thus implying the process of photoexcitation of electron carriers in the perovskite thin films is not the main limiting factor of the QE. The efficiency of the step (ii) is largely determined by the transport properties of the perovskite films. After photoexcitation the charge carriers in the films are subject to an electric field of about 3.6 V/cm and the efficiency of charge transport to the halide perovskite surface is quantified by the mobility-lifetime product (*µτ*). In halide perovskites, *µτ* is typically between 10^−2^ and 10^−4^ cm^2^/V depending on the material composition, which is on par or better than III-V semiconductors^8,28,32,33^. Moreover, transport of charge carriers in the bulk of 3D perovskite thin films is very efficient across a distance of 500 nm^10,11^. Therefore, we infer that in 3D halide perovskites the QE of the photoelectric effect is mainly limited by the step (iii), which is the efficiency of ejecting the photogenerated electrons out of the surface. The efficiency of step (iii) is limited by the recombination of electrons at surface defects and surface energy barriers for electrons^34^. The probability of surface recombination depends on the surface recombination velocity *S*, which needs to be minimized in order to achieve high QE—for example smaller than 10^4^ cm/s in GaAs electron sources^35^. Previous reports have claimed *S* values of the order of 10^3^−10^4^ cm/s in CsPbBr_3_ single crystal^36^, and values as low as 4 cm/s in MAPbBr_3_ single crystals under specific passivation conditions^37^. However, polycrystalline films might have significantly higher *S* due to the presence of grain boundaries and a higher density of surface defect states as compared to single crystals in the absence of any surface treatment. Indeed, in the FA_0.7_MA_0.25_Cs_0.05_PbI_3_ thin films, we recently demonstrated that charge collection in solar cells is limited by interface defects, which factors into the lower QE observed in these films^28^. In addition to these effects, in 2D Ruddlesden-Popper layered perovskites less effective transport of the photo-excited electrons to the film surface (*µτ* ~10^−6^–10^−5^ cm^2^/V) can also contribute to the observed lower QE, as explained in our recent work on 2D perovskite photovoltaic devices demonstrating field-dependent charge collection possibly limiting electron-hole separation^38^.

To verify that in 3D perovskites the QE is limited by the ability of electrons to escape from the perovskite surface and into the vacuum, we elucidate the impact of the Cs deposition on the emission of free electrons. The minimum photon energy for electron emission in vacuum corresponds to the work function hν = W, where *h* is the Plank constant, *ν* is photon frequency, and *W* the work function. The work functions values reported in the present paper for all pristine perovskites (around 4.0 eV), including CsPbBr_3_ (vide infra) are consistent with the work function of CsPbBr_3_ thin films reported in a recent study at 4.1 eV^39^. We may notice that these values differ from the bulk ionization energies IE = *E*_gap_ + *χ*, where *E*_gap_ is the bandgap and *χ* is the electron affinity, defined as the difference between vacuum level and conduction band minimum. Indeed, the ionization energy was measured to be around 5.8 eV in CsPbBr_3_ thin films^39^. Consistently, the pristine CsPbBr_3_ thin films in the present work yield an emission onset of 4.0 eV, far above the expected bulk ionization energy. Another surprising observation is that the photoelectron onset at 4.0 eV is also common to CsPbI_3_ and the three hybrid perovskites that were tested, before Cs activation (Fig. 2a and Supplementary Fig. 1). This result is consistent with recent reports claiming negligible dependence of the work function of perovskite thin films as a function of their compositions due to pinning of the Fermi energy, which has been explained by the similar nature of surface states in all perovskite films^39^. The good correspondence between the CsPbBr_3_ work function and the electron emission onset suggests that the activation of deep surface traps under vacuum conditions is related to the metal cation Pb at the film surface^37^. We explain the orders of magnitude increase of the QE upon Cs surface deposition by a conditioning of the thin film surfaces resulting in a change of sign of the electron affinity (Fig. 3c)^34^. Upon deposition of Cs on the perovskite surface, the Cs lose an electron, resulting in the formation of a surface dipole layer that lowers the electron affinity to a negative value^14,18,23,40^. Correspondingly, first-principles calculations explain the increase of QE upon the addition of a Cs layer onto perovskite crystals by a decrease of the work function, which may result from changes in the Fermi energy and density of states at the conduction band minimum (see section ST1 of the supplementary materials and Supplementary Fig. 3). Even though requirements for surface conditioning are relaxed in halide perovskites in comparison with III-V semiconductors^18,24^, both the inhomogeneous spatial distribution of QE (Fig. 3d) and the significantly different QE values observed in the three types of 3D perovskite films underscore the limited understanding and control of the Cs activation process in halide perovskites and is the main limiting factor for achieving higher QE.

To better understand the relationship between Cs coverage on the halide perovskite surface and QE, we performed in-situ AES measurements. The Cs coverage is quantitatively determined by calculating the ratio between Cs (49 eV) and Br (55 eV) Auger peaks (see section ST2) and plotted as a function of QE at 405 nm in Supplementary Fig. 8b. The QE continuously increased as Cs coverage increased from 0 till about 2.5 Cs/unit cell where QE reached its maximum value. This is in good agreement with our theoretical calculations, which show that two layers of Cs coating (2 Cs/unit cell) has a lower work function than one layer of Cs coating. Furthermore, deposition of more than two layers of Cs decreases the QE, similar to what has been observed on GaAs photocathodes^41^.

### **S**tability of the photoelectric effect during halide perovskite electron source operation

**Fig. 4:**
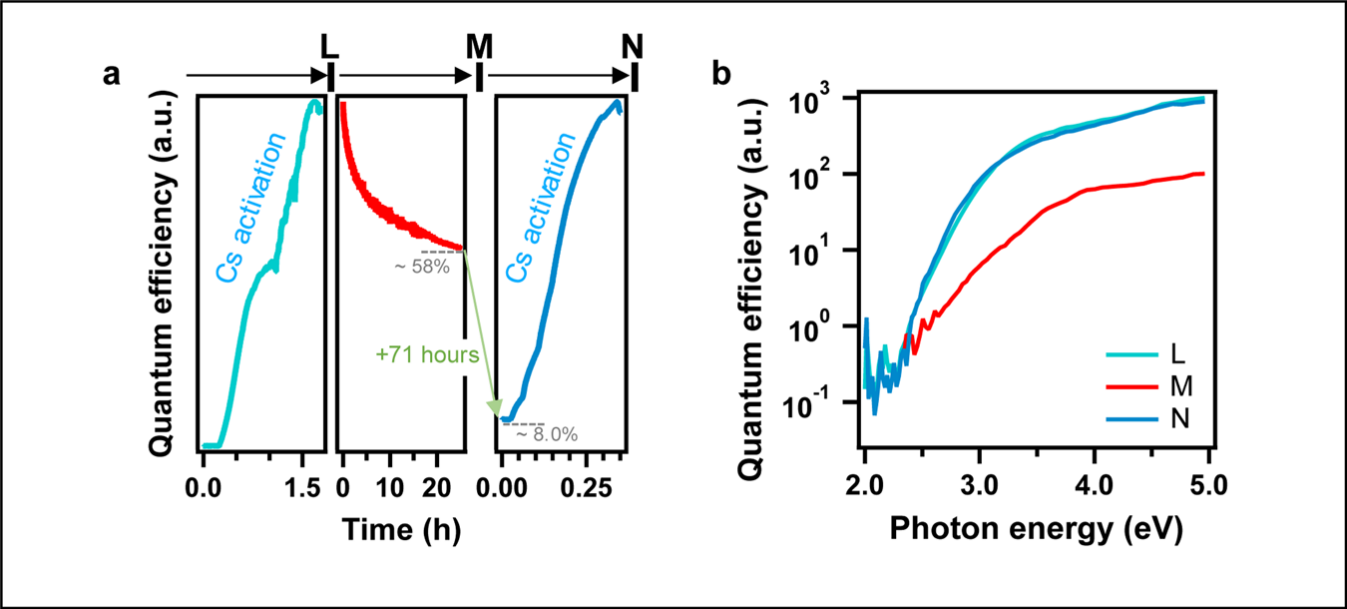
Stability of the photoelectric effect. **a** Time evolution of the quantum efficiency of the photoelectric effect in the CsPbBr3 thin films measured for continuous illumination at 3.06 eV and base vacuum of 10^-9^ torr. The left and right sub-plot correspond to Cs activation processes during which we expose the films to the Cs source and at the same time monitor the quantum efficiency (see also Supplementary Fig. 6). The central sub-plot probes monitor the efficiency degradation over 25 h. The illumination was kept until 96 h when the second Cs activation was performed. **b** Corresponding quantum efficiency spectra were taken at points L, M, and N.

Next, we investigated the stability of the photoelectric effect in the CsPbBr_3_ thin films. Figure 4a (central panel) illustrates the evolution of the QE over time as a film is continuously illuminated with light at 3.06 eV. We observed that the QE degrades to 60% of its original value after 25 h and down to 8% after 96 h. The main degradation mechanism is attributed to the surface contamination due to the presence of oxygen-based elements inside the vacuum chamber. We quantify the stability of the perovskite films by calculating the exposure in units of Langmuir (L) equivalent to the dose of a given element for the QE to decrease by 63%. We estimated the minimum values of 2.86 L, 6.75 L, and 6.28 L for H_2_O, O_2_, and CO_2_, respectively. Under similar conditions of vacuum pressure as used here (10^−9^ torr), state-of-the-art semiconductor electron sources yield lower exposures with rare exceptions such as GaN^22,25,42^. For example, about 0.05 L for H_2_O was found in a CsK_2_Sb electron source, and exposures smaller than 0.08 L were reported in a GaAs electron source with an initial QE of 12%^18,25^.

Finally, we observed that after degradation the QE of the perovskite thin films could be regenerated back to its original value (i.e. before degradation) via in-situ Cs deposition in about 1 h. Figure 4a illustrates a complete cycle starting from an initial Cs activation (left panel where the QE is continuously monitored while the films are exposed to the in-situ Cs source), followed by a decrease in the QE due to degradation during operation (central panel), and then a second Cs activation process that regenerates the perovskite thin films (right panel). The corresponding QE spectrum of the photoelectric effect taken at different time of the degradation–regeneration cycles confirms that the thin film is almost perfectly regenerated to its original QE value after the second Cs activation (Fig. 4b). It was demonstrated that the main degradation mechanism in semiconducting GaAs electron sources results from the alteration of the Cs activation layer on the surface. Indeed, exposition of the Cs surface to oxygen-based residual gas in the vacuum chamber leads to the formation of Cs–O and Cs–OH compounds, thus compromising the beneficial effect of Cs on the performances of the electron sources^26,43^. Our observation indicates no appreciable degradation of the halide perovskite films as, after degradation, we can almost fully restore the QE of our electron sources through a second Cs activation (Fig. 4a). Therefore, it is reasonable to assume that the main degradation in our electron sources takes place at the Cs surface, most likely through a mechanism similar to the one described in GaAs electron sources. The in-situ AES measurements show the Cs coverage decreased from 2.5 Cs/unit cell (maximum QE) to 2.1 Cs/unit cell after degradation (Supplementary Fig. 8b) while the relative oxygen Auger peak increased by ~30% (Supplementary Fig. 9). After adding fresh Cs on the degraded photocathode, the Cs coverage increased to 2.7 Cs/unit cell and the oxygen peak decreased to a magnitude similar to that of the originally activated surface. Moreover, we observe in Fig. 4a (left and right panels) that the Cs-activation process after degradation is about four times faster than the initial Cs-activation (from the pristine films), which is another indication that the Cs surface is only partially compromised by the oxygen-based residual gas and needs to be restored with Cs atoms.

In summary, we have demonstrated perovskite-based electron sources with a few percent quantum efficiencies, with spectral tunability other than the visible spectral range, and which can be operated for tens of hours and integrally regenerated in situ after degradation. The figures of merit reported for the photoelectric effect in halide perovskite thin films offer a tremendous opportunity to develop a disruptive electron source technology that addresses the demands for low-cost fabrication and operation, high efficiency, and spectral tunability. Furthermore, future optimization of the quality of the perovskite thin films and the Cs deposition process should lead to electron sources preforming on par with those obtained using III-V semiconductors but with orders of magnitude lower cost in terms of manufacturing and operation.

## Methods

### Thin film samples preparation

All chemicals, lead bromide (PbBr_2_), lead iodide (PbI_2_), cesium bromide (CsBr), cesium iodide (CsI), N, N-dimethylformamide (DMF), dimethyl sulfoxide (DMSO), formamidinium iodide (FAI), methylammonium iodide (MAI), butylammine (BA) were purchased from Sigma-Aldrich with the purity of 99% or higher. Equal molar of PbBr_2_ and CsPb were mixed in DMSO for 0.4 M CsPbBr_3_ solution. Equal molar of PbI_2_ and CsI was mixed in DMF for 0.6 M CsPbI_3_ solution. Both solutions were stirred for 24 h. The Ruddlelden-Popper layered perovskite solutions were prepared by dissolving the layered perovskite single crystals in DMF with 0.255 M molar concentration. The Ruddlesden-Popper layered perovskite single crystals were synthesized following a method reported previously^44–47^. PbO powder was dissolved in a mixture of 57% w/w aqueous HI solution and 50% aqueous H_3_PO_2_ by heating to boiling under constant magnetic stirring. An appropriate molar amount of MAI and BA were added to the hot solution separately. The solution was left to cool to room temperature while the perovskite crystals started to precipitate. FA_0.7_MA_0.25_Cs_0.05_PbI_3_ solution was a mix of PbI_2_, FAI, MAI, and CsI with the desired molar ratio and dissolved in DMSO with a molar concentration of 0.43 M^28^. Doped silicon substrates were cut into 1 inch^2^ and treated by oxygen plasma before spin coating. The substrates resistivity was chosen between 0.001 and 0.005 Ω·cm and their thickness about 500 µm, in order to prevent any performance limitation due to the substrates. The prepared solutions were spin coated onto the Si substrates with a speed 2000 rpm for CsPbBr_3_ and 3000 rpm for CsPbI_3_ in order to get ~200 nm thick films. The films were then annealed on a hot plate at 100 °C for 10 min. The 2D perovskite and FA_0.7_MA_0.25_Cs_0.05_PbI_3_ films were prepared by hot casting method^29,47^. All processes were conducted in an Ar filled glovebox except for substrate preparation. The samples were transported in a sealed vial filled with argon and loaded into the vacuum chamber load lock with minimum exposure to air (less than 5 min). Inside the load lock, only the CsPbBr_3_and CsPbI_3_ thin films were baked out at 350 °C for 24 h to clean the solvent residue and absorbed moistures; the hybrid halide perovskite films were not backed to prevent degradation due to temperature. After the samples were cooled down, they were loaded into the main chamber with a base pressure of about 10^−9^ torr and with residual gases partial pressures smaller than 10^−10^ torr (H_2_O 1.59 × 10^−11^ torr, O_2_ 3.75 × 10^−11^ torr, CO_2_ 3.49 × 10^−11^ torr). The vacuum chamber was analyzed by a Stanford Research System Residual Gas Analyzer.

### Photoelectric effect measurement

For photoexcitation of the thin films inside the vacuum chamber, we used the monochromatic light generated by a Newport 150 W Xenon lamp coupled with a monochromator equipped with a 300 l/mm grating and long-pass filters; the range of monochromatic light available for the QE spectra was 4.96 to 1.55 eV (250 to 800 nm). The light was modulated at 400 Hz by a chopper and then directed into the chamber by a mirror and through a quartz window at 45° incident angle. The light beam was illuminating about 10 mm^2^ of the thin film surface if not mentioned otherwise, and the light power at the sample position was in the range 38−50 µW. In this regime, the QE of the films does not show appreciable changes as a function of light intensity, in order to keep surface charging effects at a minimum (Supplementary Fig. 5). For mapping of the QE, the light beam from a 405-nm laser was scanned onto the thin film surface using a stirring mirror and the photocurrent was acquired at each scanned point. The anode was placed 25 mm away from the film substrates, and biased at +90 V against the doped Si substrate using DC batteries in order to minimize electronic noise. The chamber was connected to the anode to eliminate any possible chamber emission. In our setup, 90 V was tested to be high enough to collect all photoelectrons (Supplementary Fig. 4). A 100 kΩ resister was connected in series with the cathode to convert the photocurrent into voltage. The circuit capacitance was 2.7 nF, corresponding to a time constant of 0.27 ms, much faster than the light modulation period (2.5 ms). The voltage was measured by an SR830 lock-in amplifier with a time constant between 0.3 s and 10 s depending on the magnitude of the signal. All measurements were performed at ambient temperature.

### Cs deposition

For the process of Cs activation, we applied current to a 1 cm long SAES Cs dispenser as a Cs deposition source. Prior to deposition, the source was first degassed in an antechamber at a current of 4 A. The source was then placed in front of the sample with a 2.5–5 cm distance. The light source was placed at 405 nm to monitor the photocurrent from the photoelectric effect. The current was slowly ramped up at a rate of 0.1 A/min until the photocurrent started to increase, typically around 4.5 to 5 A. The source current was either maintained or further increased to maximize the photocurrent, just before the photocurrent started to drop meaning in that case that the deposited layer of Cs onto the thin film surface was too thick (Supplementary Fig. 6). The Cs source was then retracted from the deposition position and turned off. An energy-dependent scan was performed immediately after the Cs deposition to measure the QE spectrum. From our observations, it is reasonable to assume that the Cs layer on the film’s surface is limited to at most a few monolayers of Cs and its thickness is relatively inhomogeneous due to the roughness of the films. This is supported by several evidences: the lack of electron emission from the Cs layer, the low sticking coefficient between Cs atoms and the low melting temperature (28.4 °C), and the rapid degradation of the QE of halide perovskite sources for thicker Cs layers (Supplementary Fig. 6) consistent with observations reported in Cs activated GaAs electron sources.

### Auger electron spectroscopy

The Auger spectra were obtained with a SPECS Phoibos 150 hemispherical energy analyzer and a five channel multi channeltron detector (MCD-5). The electron gun was set at 3 keV energy and 1 µA current. The area of the electron beam was ~1 mm^2^. The AES UHV chamber was connected to the Cs deposition chamber through a sample transfer arm.

## Supplementary information

Supplementary Information

## Data Availability

The data that support this study within the paper and supplementary materials are available from the corresponding authors upon request.
