# Supplementary material for: Highly efficient photoelectric effect in halide perovskites for regenerative electron sources
Source: Nat Commun. 2021 Jan 29;12:673. doi: 10.1038/s41467-021-20954-6 (PMC7846809; doi:10.1038/s41467-021-20954-6)
Supplement: Supplementary file 1 — Supplementary Information [file 41467_2021_20954_MOESM1_ESM.pdf]

Supplementary Information for

Highly efficient photoelectric effect in halide perovskites for regenerative electron sources

Fangze Liu[1], Siraj Sidhik[2], Mark A. Hoffbauer[1], Sina Lewis[1], Amanda J. Neukirch[1], Vitaly Pavlenko[1], Hsinhan Tsai[1], Wanyi Nie[1], Jacky Even[3], Sergei Tretiak[1], Pulickel M. Ajayan[4], Mercouri G. Kanatzidis[5, 6, 7], Jared J. Crochet[1], Nathan A. Moody[1], Jean-Christophe Blancon[2]* and Aditya D. Mohite[2]*

[1]Los Alamos National Laboratory, Los Alamos, New Mexico 87545, USA.

[2]Department of Chemical and Biomolecular Engineering Rice University, Houston, Texas 77005, USA.

[3]Fonctions Optiques pour les Technologies de l'Information, FOTON UMR 6082, CNRS, INSA de Rennes, 35708 Rennes, France.

[4]Department of Material Science and Nanoengineering Rice University, Houston, Texas 77005, USA.

[5]Department of Chemistry, Northwestern University, Evanston, Illinois 60208, USA.

[6]Department of Materials Science and Engineering, Northwestern University, Evanston, Illinois 60208, USA.

[7]Argonne-Northwestern Solar Energy Research (ANSER) Center, Northwestern University, Evanston, Illinois 60208, USA.

***Correspondence: jrb21@rice.edu & adm4@rice.edu**

Supplementary Note 1 – Modelling of the effect of Cs activation

To gain insight into the mechanism of Cs activation in the halide perovskite materials, we investigated by density function theory (DFT) the effect of Cs coating onto the [001] and [110] surface of both $CsPbBr_3$ and $CsPbI_3$. We note that $CsPbBr_3$ is orthorhombic phase at room temperature that is slightly different from cubic phase by an octahedral tilting. Upon heating, the orthorhombic (101) and (202) planes converts into cubic (001) and (002) planes with a minor shift of 0.2 degree. For simplicity, we use the ideal cubic phase in DFT calculations. This phase transition does not significantly affect the bandgap or the surface structure of $CsPbBr_3$. We further checked that computed work functions with Cs coating do not depend strongly on the surface orientation.

DFT calculations were performed with the Vienna Ab Initio Simulation Package (VASP) using the projector-augmented wave method.[1-3] We found that the optimal slab thickness was 5 polyhedral (108 atoms and ~29.2 Å for $CsPbBr_3$, ~31.4 Å for $CsPbI_3$) for a balance between computational cost and accuracy. The surface of the $CsPbX_3$ slabs was modeled in the three-dimensional periodic-boundary-condition simulation with a vacuum gap in the direction perpendicular to the surface. An energy cutoff of 480 eV was chosen to generate the plane-wave basis sets and a gamma-centered (6x6x1) k-points mesh was used to sample the reciprocal space for the [001] surface. A value of 30 Å of vacuum was chosen to minimize the interaction between periodically repeated slabs. Three cases were investigated for each $CsPbX_3$ slab: a pristine surface, one layer of Cs on the surface, and two layers of Cs on the surface, as shown in Supplementary Fig. 3. Dipole corrections normal to the surface were included to numerically correct the electrostatic potential for spurious dipolar-dipolar contributions between slabs due to the small inter-slab distances. Here we precise that the Fermi energy in DFT refers to the highest occupied electron level at zero Kelvin temperature and the vacuum level to the flat part of the dipole corrected electrostatic potential in the vacuum close to the slab surface. Therefore, and due to limitations of computational resources to simulate such large systems, the analysis of DFT computed work functions (*i.e.* the difference between the Fermi energy and vacuum level) should be made carefully; however, DFT offers qualitative insights into the basic trends and the changes of the density of electronic states with respect to the vacuum level induced by Cs coating at the surface.

Supplementary Table 1 summarizes the change of DFT computed work function of $CsPbX_3$ [001] and [110] surfaces before and after Cs coating. For the [001] surface, the calculated work function values of the $CsPbX_3$ materials with pristine surfaces are between 4.3 and 4.7 eV, yielding reasonable agreement with experimental values.[4] Upon the addition of a mono- or double-layer of Cs onto the materials surface, the work function of $CsPbBr_3$ and $CsPbI_3$ decrease drastically to values between 1.8 and 2.2 eV. Similar results are observed for the [110] surface. These results correspond reasonably well with the decrease of the onset energy of the photoelectric effect measured and reported in Fig. 2a. To gain deeper understanding the origin of the lowered work function, we calculated partial density of states DOS (Supplementary Fig. 3) of pristine and Cs coated $CsPbBr_3$ surface. For pristine $CsPbBr_3$, shown in Supplementary Fig. 2a, the DFT Fermi energy corresponds to the maximum of the valence band and Pb dominates the DOS at the conduction band minimum. Upon addition of a Cs layer onto the $CsPbBr_3$ surface, as shown in Supplementary Fig. 2b, the Fermi level increases by about 1.6 eV thus falling into the conduction

band, and the conduction band DOS becomes dominated by electronic states related to Cs. Both effects contribute additively to the lowering of the work function and provide a qualitative explanation for the increase of the QE and lowering of the onset energy of the photoelectric effect upon Cs activation.

Supplementary Note 2

To quantitatively determine the Cs coverage, we monitored the peak ratio between Cs (49 eV) and Br (55 eV) in the Auger spectra N(E) as a function of Cs coverage, see example in Supplementary Fig. 8c. This approach is justified as the electron inelastic mean free path for energy between 50 and 500 eV is in the range of 5-10 Å. [5] Considering the lattice constant of 5.87 Å, [6] the probing depth of AES around 50 eV is slightly less than one unit cell of $CsPbBr_3$. In addition, studies on $CsPbBr_3$ nanocube, nanoplatelets and quantum dots nanocrystals[7-9] show the $CsPbBr_3$ crystal surface is preferably terminated by CsBr with possible Cs vacancies. Therefore, the surface stoichiometry within the AES probing depth (5 Å) is between Cs_2PbBr_4 (one unit cell) and $CsPbBr_3$ (top two layers of atoms). The Cs to Br ratio is plotted as a function of QE at 405 nm in Supplementary Fig. 8b, and curve fittings for Cs and Br peaks are plotted in Supplementary Fig. 8c. The initial Cs/Br ratio ($R_{Cs/Br}$) before Cs deposition is 2.5. Assuming each unit cell within the 5 Å probing contains 1.5 Cs atoms (average of Cs2PbBr4 and CsPbBr3), we estimate the deposited Cs coverage by$\left(R_{Cs/Br}/2.5 - 1\right) \times 1.5$. We note that our calculation may overestimate the Cs coverage since more Cs is deposited, the deposited Cs will contribute more to the Auger signal.

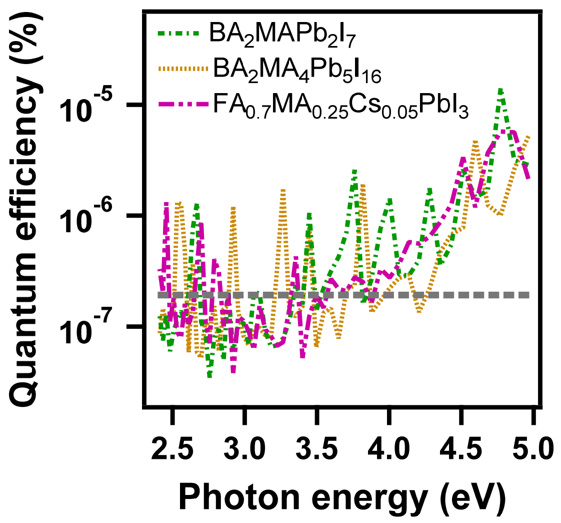

Supplementary Fig. 1. Photoelectric quantum efficiency spectra of the hybrid halide perovskite thin films before Cs activation. The horizontal gray dashed line is guide for the eyes indicating the noise detection level for this experiment.

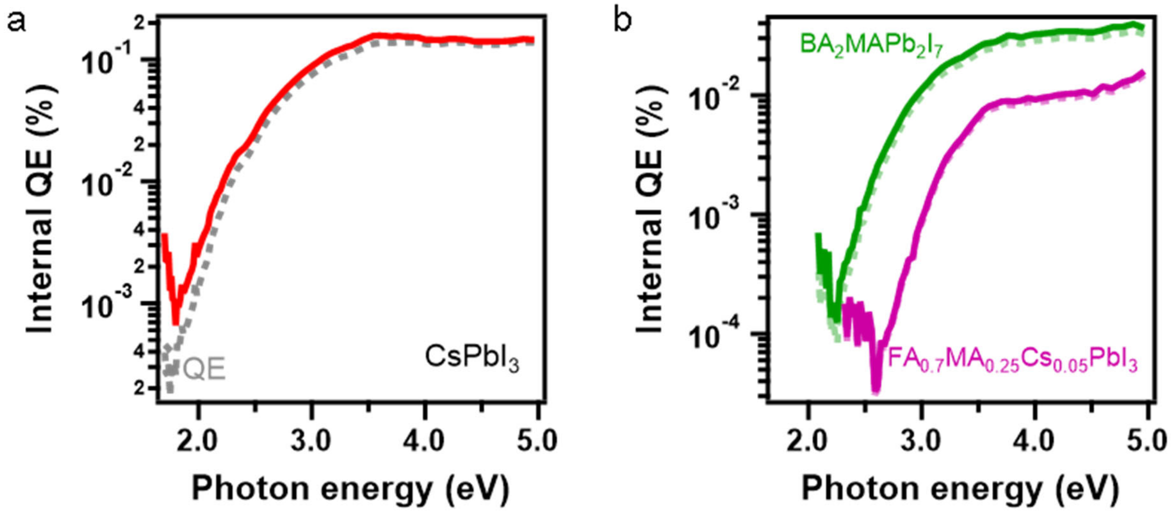

Supplementary Fig. 2. Internal quantum efficiency spectra of the (a) $CsPbI_3$ and (b) hybrid halide perovskite thin films. The dashed curves are the corresponding quantum efficiency of the films.

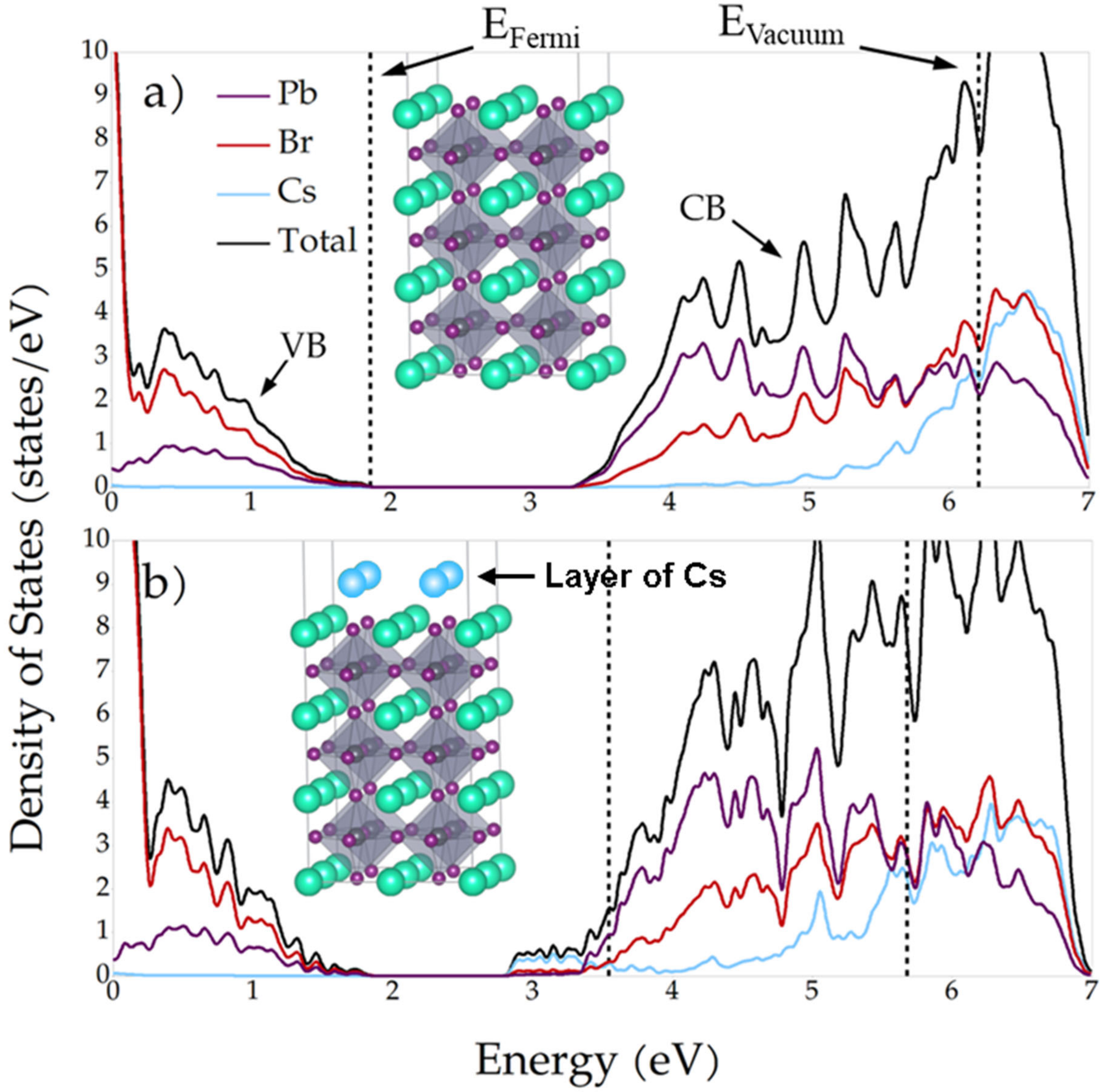

Supplementary Fig. 3. Calculated density of states of CsPbBr3 [001] surface without (a) and with (b) monolayer Cs coating. The total density of states is black and the partial density of states of are plotted as Pb (purple), Br (red) and Cs (blue).

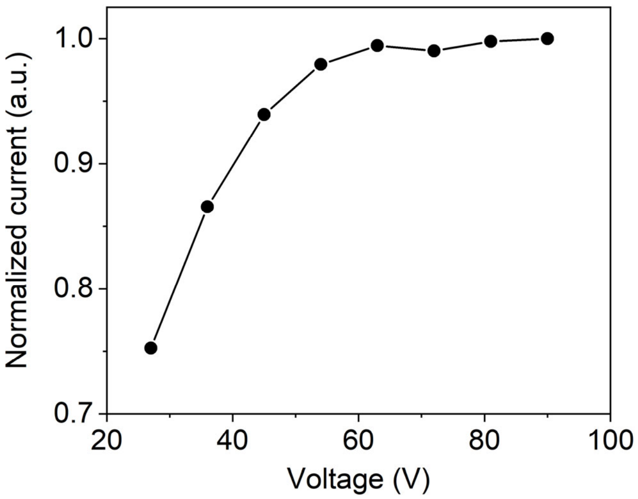

Supplementary Fig. 4. Dependence of the photocurrent from a CsPbBr3 thin film on the potential difference between the film substrates and counter anode. The film was excited with a 300-nm monochromatic light. The saturation of the photocurrent above 60 V demonstrates that 90 V was adequate to collect all electrons emitted from the thin films.

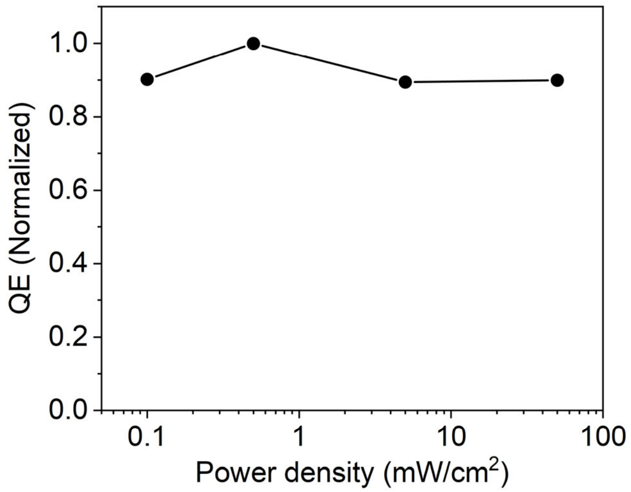

Supplementary Fig. 5. Excitation light intensity dependence of the quantum efficiency in $CsPbBr_3$ thin films. Light excite the films with 405 nm and spot size of 1 mm^2.

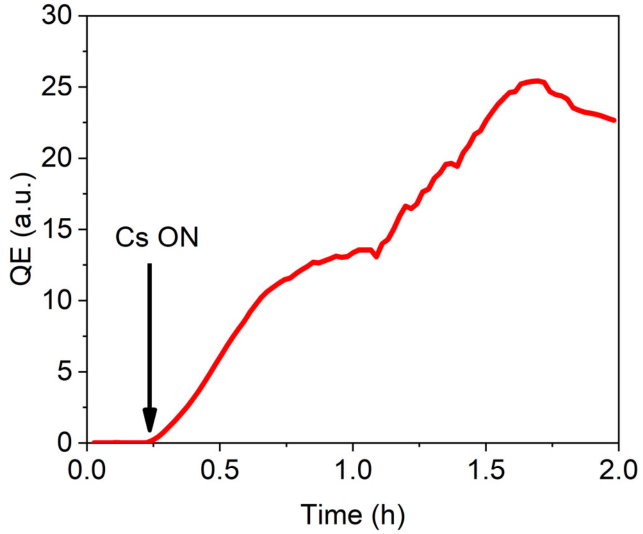

Supplementary Fig. 6. Typical evolution of the quantum efficiency of halide perovskite thin films as a function of the Cs deposition time on the films surface. The Cs activation to maximum performance of the photoelectric effect in halide perovskite thin films is about one hour, beyond which the Cs surface layer becomes too thick and degrades the quantum efficiency of the films.

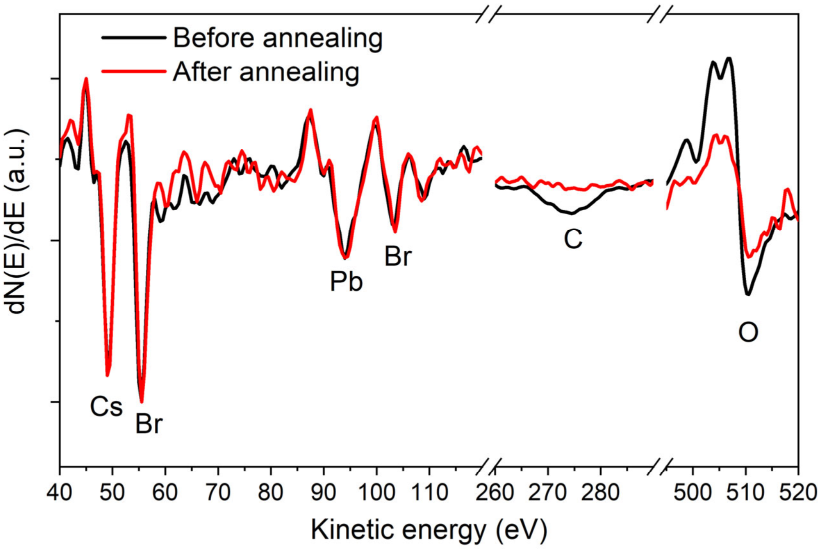

Supplementary Fig. 7. **Comparison of Auger spectra of $CsPbBr_3$ film before and after UHV annealing.** The magnitudes of characteristic Auger peaks from Cs (49 eV), Br (55 eV) and Pb (97 eV) remained unchanged after UHV annealing, indicating the annealing didn’t change the surface stoichiometry.

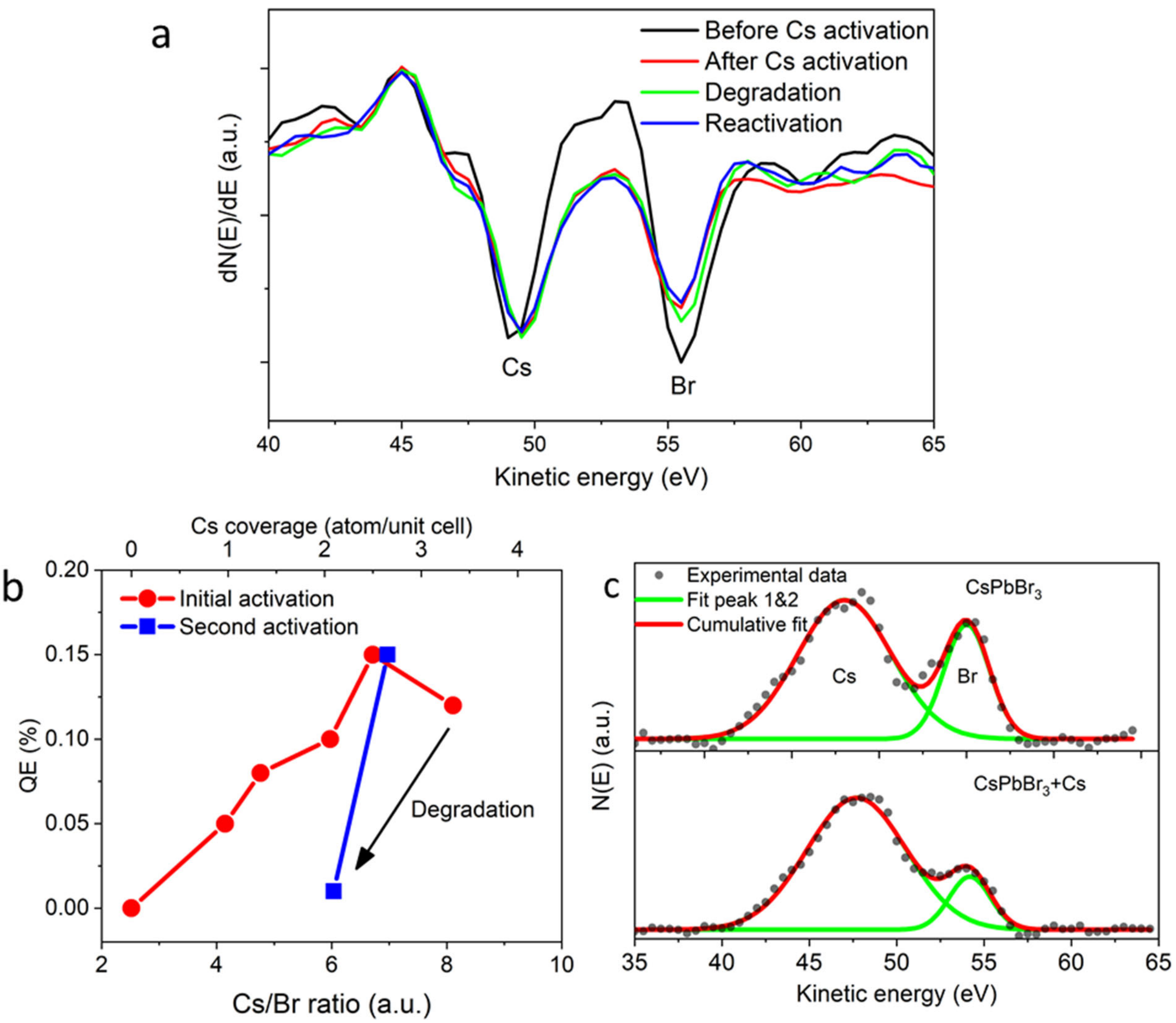

Supplementary Fig. 8. **Auger peak analysis for Cs and Br.** a, evolution of differential Auger spectra during Cs activation process. b, evolution of Auger peak ratio between Cs and Br during Cs deposition and degradation processes. c, sample peak fitting for pristine $CsPbBr_3$ and Cs coated $CsPbBr_3$.

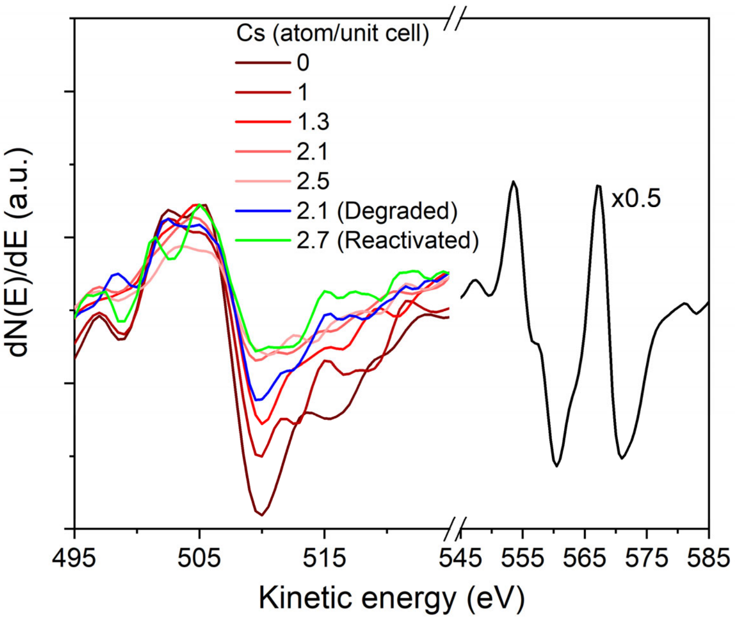

Supplementary Fig. 9. The evolution of O KLL transition at 510 eV during Cs deposition. The spectra are normalized to the Cs MNN transition at 572 eV, which is shown for reference. The Cs Auger peak amplitude is multiplied by 0.5.

**Materials**	**$CsPbI_3$**			**$CsPbBr_3$**		
**Cs coating**	None	1 layer	2 layers	None	1 layer	2 layers
**[001]**	4.695	2.128	1.804	4.358	2.133	2.030
**[110]**	3.879	2.165	2.034	3.878	2.283	2.084

Supplementary Table 1. Calculated work function of the [001] and [110] surfaces of $CsPbX_3$.

Supplementary References

1. Kresse, G. & Furthmüller, J. Efficiency of ab-initio total energy calculations for metals and semiconductors using a plane-wave basis set. *Comput. Mater. Sci.* **6**, 15–50 (1996).
2. Kresse, G. & Joubert, D. From ultrasoft pseudopotentials to the projector augmented-wave method. *Phys. Rev. B* **59**, 1758–1775 (1999).
3. Kresse, G. & Furthmüller, J. Efficient iterative schemes for ab initio total-energy calculations using a plane-wave basis set. *Phys. Rev. B* **54**, 11169–11186 (1996).
4. Endres, J. *et al.* Valence and conduction band densities of states of metal halide perovskites: a combined experimental–theoretical study. *J. Phys. Chem. Lett.* **7**, 2722–2729 (2016).
5. Tanuma, S., Powell, C.J. & Penn, D.R. Calculations of electorn inelastic mean free paths. II. Data for 27 elements over the 50–2000 eV range. *Surf. Interface Anal* **17**, 911-926 (1991).

6. Murtaza, G. & Ahmad, I. First principle study of the structural and optoelectronic properties of cubic perovskites $CsPbM_3$ (M=Cl, Br, I). *Physica B: Condensed Matter* **406**, 3222–3229 (2011).

7. Brennan, M. C., Kuno, M. & Rouvimov, S. Crystal Structure of Individual $CsPbBr_3$ Perovskite Nanocubes. *Inorg. Chem.* **58**, 1555–1560 (2019).

8. Bertolotti, F. et al. Crystal Structure, Morphology, and Surface Termination of Cyan-Emissive, Six-Monolayers-Thick $CsPbBr_3$ Nanoplatelets from X-ray Total Scattering. *ACS Nano* **13**, 14294–14307 (2019).

9. Chen, Y. *et al.* Surface Termination of $CsPbBr_3$ Perovskite Quantum Dots Determined by Solid-State NMR Spectroscopy. *J. Am. Ch*
